# Maxillectomy Guided by 3D Printing Versus Conventional Surgery for Patients with Head and Neck Cancer

**DOI:** 10.3390/cancers17010140

**Published:** 2025-01-04

**Authors:** Sung Yool Park, Sung Ha Jung, Anna Seo, Hakjong Noh, Hwansun Lee, Hyo Jun Kim, Younghac Kim, Man Ki Chung, Han-Sin Jeong, Chung-Hwan Baek, Young-Ik Son, Nayeon Choi

**Affiliations:** 1 Department of Otorhinolaryngology-Head and Neck Surgery, College of Medicine, Busan Paik Hospital, Inje University, Busan 47392, Republic of Korea; 2 Department of Otorhinolaryngology-Head and Neck Surgery, Samsung Medical Center, Sungkyunkwan University School of Medicine, 81 Irwon-ro, Gangnam-gu, Seoul 06351, Republic of Korea; 3 Seeann Solution, Co., Ltd., Incheon 21984, Republic of Korea; 4 Department of Otorhinolaryngology-Head and Neck Surgery, Kangpook Samsung Hospital, Sungkyunkwan University School of Medicine, Seoul 06351, Republic of Korea

**Keywords:** 3D printing, maxillectomy, resection margin, oncologic outcome

## Abstract

Background: This study evaluates the impact of three-dimensional (3D) printing-guided maxillectomy compared with conventional maxillectomy on surgical precision and oncological outcomes in patients with head and neck cancer. Materials and Methods: A retrospective analysis was conducted on 42 patients undergoing maxillectomy (16 in a 3D printing-guided group and 26 in a conventional group). Patient demographics, tumor characteristics, and outcomes were compared. Survival outcomes were analyzed using the Kaplan–Meier method. Results: The 3D printing group showed higher rates of negative resection margins (81.3% vs. 76.9%) compared with the conventional group and a trend toward improved 5-year local recurrence-free survival (87.5% vs. 58.7%, respectively) and overall survival (84.4% vs. 70.1%, respectively). However, the differences were not statistically significant. Conclusions: Maxillectomy guided by 3D printing may offer enhanced surgical precision and improved local control in patients undergoing head and neck cancer surgeries. Further research with larger cohorts is necessary to confirm these findings.

## Simple Summary

This study compares 3D printing-guided maxillectomy with conventional maxillectomy in terms of surgical precision and oncological outcomes in head and neck cancer patients. A retrospective analysis of 42 patients (16 in the 3D printing-guided group, 26 in the conventional group) was conducted, comparing demographics, tumor characteristics, and oncologic outcomes. The 3D printing-guided group showed slightly higher rates of negative resection margins (81.3% vs. 76.9%) and trends toward superior 5-year local recurrence-free survival (87.5% vs. 58.7%) and overall survival (84.4% vs. 70.1%), although differences were not statistically significant. Three-dimensional printing-guided maxillectomy demonstrates potential improvements in surgical precision and local control, warranting further validation with larger studies.

## 1. Introduction

Sinonasal malignancy is a rare tumor that accounts for less than 5% of head and neck tumors, often presenting with minimal or no early symptoms [1,2,3]. Nasal obstruction, rhinorrhea, epistaxis, and facial fullness may occur but all are easy to confuse with benign conditions such as allergic rhinitis and chronic sinusitis, leading to delays in diagnosis. It is more common for such tumors to be discovered at the T3 stage or higher [4,5].

Surgery is a potentially curative option for resectable sinonasal cancers, typically aiming for complete resection and negative margins. In cases of locally advanced stage (T3 or higher), excising the tumor through endoscopic surgery can be difficult, resulting in a preference for open maxillectomy. However, even in open maxillectomy, tumors may reside within complex three-dimensional bony structures, and a limited surgical field and approach can complicate achievement of negative resection margins [6,7,8,9].

Three-dimensional printing has revolutionized healthcare by enabling patient-specific therapies over traditional, generalized protocols. This shift to precision medicine through 3D-printing has led to the creation of tailored treatment regimens. Such advances have been particularly useful in oral and maxillofacial surgery, in which precision medicine plays an integral role in practice [10,11,12,13,14]. One of the most prominent applications within oral and maxillofacial surgery has been in mandibular reconstruction, in which the benefits of improved cosmetic and functional outcomes, reduced surgical times, and lower complication rates of 3D printing are well documented [15,16,17,18,19,20]. However, evidence regarding the oncological benefits of 3D printing in the context of maxillectomy is lacking. This study evaluates the impact of maxillectomy guided by 3D printing compared with conventional maxillectomy on surgical precision and oncological outcomes in patients with head and neck cancer.

## 2. Materials and Methods

### 2.1. Study Design and Patients

This retrospective review was conducted at Samsung Medical Center, a tertiary healthcare center, and included 42 patients treated for maxillary sinus carcinoma from January 2017 to March 2024. Patients were allocated into two maxillectomy groups: a 3D printing-guided group (*n* = 16) and a conventional group (*n* = 26). The patient demographics, tumor characteristics, and treatment outcomes of the two groups were analyzed.

### 2.2. Ethical Statement

This study was approved by the Institutional Review Board of Samsung Medical Center (approval number 2024-11-050-001) and was performed in accordance with the principles of the Declaration of Helsinki. We ensured strict confidentiality and privacy protection of participant data by anonymizing any identifiable information and implementing robust data security measures.

### 2.3. 3D Printing Surgical Guidance Design

For this study, we used a special software called Reconeasy-3D (Version: 1.23.712; Web Link: https://www.seeann.co.kr/; access date: 13 December 2023) from SEEANN Solution to create custom 3D printed surgical guides. This software is approved as a medical device by the National Institute of Medical Device Safety Information (NIDS). By analyzing the medical imaging data (DICOM files) from each patient, we were able to design precise surgical guides tailored to each case, ensuring they worked consistently well for all patients in the study.

The materials used for the surgical guides included AMB10 and titanium, both of which are safe for medical use. These materials passed important safety tests to ensure they do not cause harmful reactions in the body, such as irritation or sensitivity.

To print these guides, we used advanced 3D printers. One type of printer uses light to create the guide layer by layer (Digital Light Processing, or DLP), while another uses powdered metal fused together with a laser (Powder Bed Fusion, or PBF). Both printers are excellent for producing strong, safe materials that can be used in surgery.

### 2.4. 3D Virtual Simulation and Surgical Technique

All surgeries were performed by two experienced surgeons and conducted by the same surgical team to ensure consistency in the procedures. Maxillectomy was performed using a Weber–Ferguson incision, with extensions made as needed to ensure adequate exposure of the tumor. The bone cutting lines were determined based on the 3D printing surgical planning and rapid prototype model in the 3D printing-guided maxillectomy group and based on physical examination and images in the conventional maxillectomy group. The maxillectomy was then executed using a reciprocating saw and curved osteotome.

**Figure 1:**
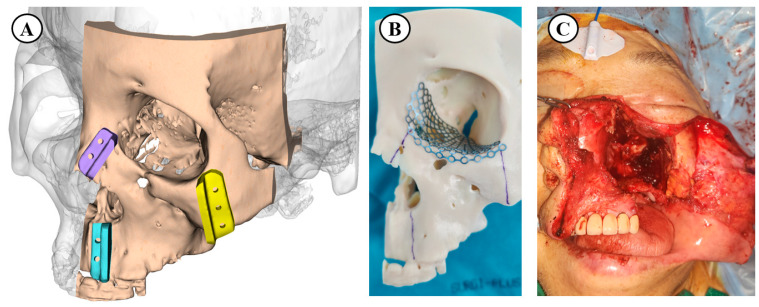
Example of 3D printing guidance design using Reconeasy-3D Software. (**A**) Preoperative virtual simulation created using DICOM data extracted from computed tomography (CT) and magnetic resonance imaging (MRI), virtually marking the areas for osteotomy guidance (**B**) The rapid prototype 3D model of the maxilla, displaying osteotomy lines to guide tumor resection and a prefabricated orbital mesh plate customized for the orbital floor defect, prepared for precise anatomical restoration during surgery. (**C**) Intraoperative view showing maxillectomy performed according to the preoperative plan created with 3D printing.

**Figure 2:**
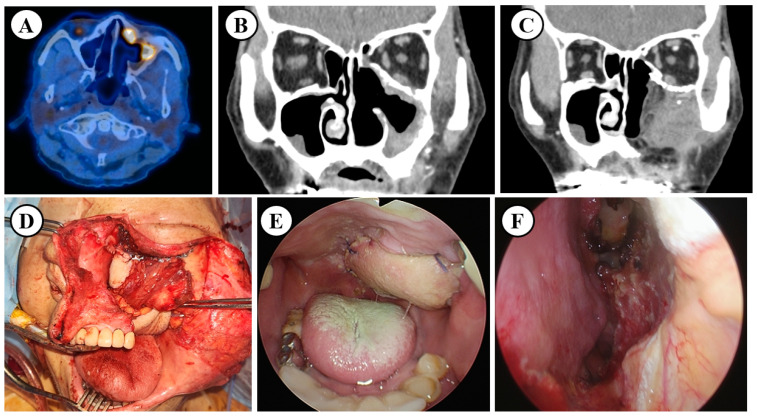
Clinical images of 79 years old male who had 3D printing-guided salvage maxillectomy after the failure of definitive chemoradiation. (**A**) PET-CT revealed maxillary sinus cancer in anterolateral wall of sinus. (**B**) Preoperative CT coronal image showed maxillary sinus cancer involving orbital inferior wall, inferolateral wall of maxillary sinus. (**C**) Postoperative CT image revealed well-reconstructed orbital plated and anterolateral thigh free flap at 3 months after the surgery. (**D**) Intraoperative image of maxillectomy defect and reconstruction with anterolateral thigh free flap and prefabricated orbital mesh plate. (**E**,**F**) Endoscopic image of hard palate and nasal cavity reconstructed by anterolateral free flap at 3 months post operation.

In the 3D printing-guided group, preoperative virtual simulations were created from DICOM data extracted from computed tomography (CT) and magnetic resonance imaging (MRI) (Figure 1A). The data were then converted into a 3D model, and a rapid prototype of the maxilla was printed. Preoperative osteotomy lines marked on this model indicated the extent of tumor invasion. In some patients, a prefabricated orbital mesh plate was designed based on this model to ensure precise anatomical restoration during surgery (Figure 1B). This approach was used for patients with complex tumor involvement, including the orbital floor and adjacent structures (Figure 1C). The extent of surgery for patients in the conventional group was determined based on tumor extent and Brown’s classification of maxillectomy (Table 1). Patients in the 3D printing-guided group underwent maxillectomy with the aid of the prefabricated 3D model and custom guides to optimize bone resection and reconstruction. For reconstruction, a combination of free flaps, including the anterolateral thigh flap, and prefabricated mesh plates was used as indicated in Figure 2.

### 2.5. Postoperative Management and Follow-Up

Postoperative management was consistent across the two groups. All patients were monitored in the intensive care unit with free flap monitoring until day 1 post operation. Postoperative pain was managed with multimodal analgesics during the admission period. Total parenteral nutrition (TPN) was administered for three days after the surgery and patients had clear water sips during these periods. After the confirmation of well healing of wound margin, patients started a soft diet.

Following a reduction in exudate, Barovac drains were removed, and patients were discharged once they were able to tolerate a soft diet. For follow-up, all patients underwent regular clinical examinations and imaging according to 2024 National Comprehensive Cancer Network guidelines. Routine imaging included neck CT and chest scans, with MRI or positron emission tomography–CT based on clinical indications every 3 to 6 months for up to 5 years postoperatively. Oncological outcomes, including local recurrence, regional recurrence, distant metastasis, and overall survival, were recorded.

### 2.6. Statistical Analyses

Demographic and clinicopathological characteristics were summarized using descriptive statistics. Distribution variations among the patient groups were evaluated using Pearson’s chi-square tests for categorical variables and Mann–Whitney U tests for continuous variables. To compare oncological outcomes between the two groups, analyses of cancer control (including local and overall recurrence) and overall survival were conducted. Five-year local recurrence–free survival, overall recurrence–free survival, and overall survival rates were estimated using Kaplan–Meier analysis. Kaplan–Meier plots were used to illustrate event outcomes, and a log-rank test was applied to compare survival curves. Statistical significance was set at a *p*-value < 0.05. All statistical analyses were performed in SPSS software (SPSS, Version 26, Chicago, IL, USA).

## 3. Results

### 3.1. Characteristics and Surgical Details

Patient characteristics and surgical details are summarized in Table 1. There were no significant differences in gender distribution (*p* = 0.055) or age (*p* = 0.092) between the two groups. Squamous cell carcinoma (SCC), which accounted for 66.7% of all cases, was less prevalent in the 3D printing-guided group (56.3%) compared with the conventional group (73.1%), but the difference was not statistically significant (*p* = 0.532). Both groups exhibited a higher prevalence of advanced-stage (T3 and T4) tumors, with the 3D printing-guided group showing a higher proportion of T4 tumors (43.8%) compared with 26.9% in the conventional group (*p* = 0.453). No significant differences were noted in the distribution of N stages, with more patients at the N0 stage in the 3D printing-guided group (100% vs. 84.6%; *p* = 0.437).

When using Brown’s classification to describe the extent of maxillectomy, type IIIb—resection of the orbital floor while preserving its contents—was more common in the 3D printing-guided group at 43.8% compared to 38.5% in the conventional group. Other types, such as IId and IVb, showed no significant variations (*p* = 0.543). The predominant reconstructive method was the anterolateral thigh flap, which was used more frequently in the 3D printing-guided group (62.5% vs. 57.7%). Skin grafting was less common in the 3D printing-guided group (12.5% vs. 23.1%), with reconstructive options showing no significant differences (*p* = 0.523).

### 3.2. Treatment Outcomes

Treatment outcomes are summarized in Table 2. Negative resection margins were achieved in 78.6% of the cohort, with the 3D printing-guided group slightly but not significantly higher at 81.3% compared with 76.9% in the conventional group (*p* = 0.720). Lymphovascular invasion (LVI) was present in 68.3% of all patients, with a higher LVI incidence in the 3D guided-printing group (80.0%) compared with the conventional group (61.5%). Perineural invasion (PNI) was observed in 61.0% of the entire cohort, with no significant difference between the groups (60.0% in the 3D printing-guided group vs. 61.5% in the conventional group).

Initial treatment strategies varied slightly; 71.4% of patients received combined surgery and adjuvant radiotherapy, compared to 68.8% of the 3D printing-guided group and 73.1% of the conventional group. The remaining patients were treated with surgery alone (31.3% of the 3D printing-guided group vs. 26.9% of the conventional group; *p* = 0.370).

Local recurrence, which occurred in 21.4% of patients, was lower in the 3D printing-guided group at 12.5% compared with 26.9% in the conventional group (*p* = 0.240). Rates of regional recurrence and distant metastasis were low and similar between the groups. Death occurred in 16.7% of the cohort, with 12.5% of the 3D printing-guided group dying compared with 19.2% of the conventional group; however, these differences were not significant.

**Figure 3:**
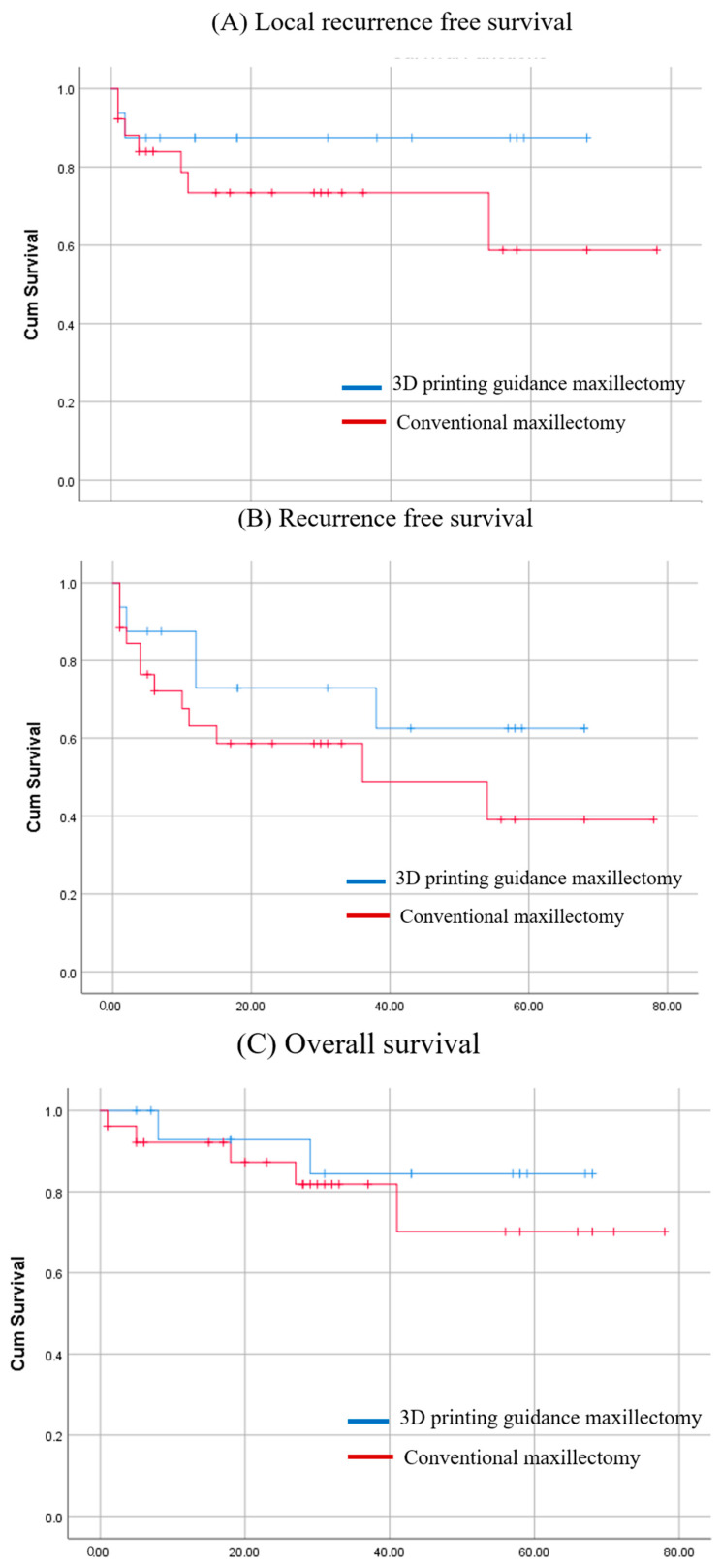
Kaplan–Meier survival plot with log-rank test between the 3D printing-guided maxillectomy group and conventional maxillectomy group. (**A**) Local recurrence free survival (*p* = 0.236); (**B**) overall recurrence free survival (*p* = 0.233); (**C**) overall survival (*p* = 0.435).

Survival analysis showed the following outcomes: Local recurrence-free survival was higher in the 3D printing-guided group at 87.5% compared with 58.7% in the conventional group (*p* = 0.236). Overall recurrence-free survival also favored the 3D printing-guided group, with rates of 62.5% versus 39.1% in the conventional group (*p* = 0.233). Similarly, overall survival was higher in the 3D printing-guided group at 84.4%, against 70.1% in the conventional group (*p* = 0.435). Kaplan–Meier survival curves comparing local recurrence-free, overall recurrence–free, and overall survival showed a trend toward improved outcomes in the 3D printing-guided group, although none of differences were significant (Figure 3).

## 4. Discussion

The treatment of locally advanced sinonasal cancer includes various approaches such as surgery, upfront concurrent chemoradiation, and neoadjuvant chemotherapy. Surgery is generally the preferred treatment for optimal oncological outcomes, unless vital structures such as the orbit or brain are involved. Invasion of these areas significantly increases morbidity and may render surgery unfeasible [6,7,8,9]. In addition, as reported previously, the overall five-year survival rate remains low. Specifically, SCC, which accounts for approximately 60% of sinonasal cancer cases, has a reported five-year overall survival rate of approximately 50% [8,21,22].

The most common cause of treatment failure is local recurrence, highlighting the importance of local control for overall survival [23,24,25,26,27]. A study of 168 patients from 1986 to 2006, reported a local failure rate of 30% and a five-year local control rate of 62% [26]. Similarly, when 127 patients were treated with curative intent from 1976 to 2003, a local failure rate of 42.5% and a five-year local control rate of 53% were reported [27].

The significance of resection margins in predicting prognosis is well-documented [28]. An analysis of data from 7,808 patients showed that sinonasal SCC tumors with macroscopically involved margins had prognoses similar to those managed with upfront nonsurgical strategies, suggesting a critical role of negative or microscopically involved margins for improved overall survival [29]. An analysis of 239 patients treated for sinonasal mucoepidermoid carcinoma found that surgeries with clear margins resulted in superior outcomes compared with those with positive margins, and that adjuvant radiotherapy conferred a survival benefit only for patients who underwent complete resection [30].

Obtaining negative resection margins during maxillectomy poses significant challenges. The complex 3D bony structure makes it difficult to visualize the tumor boundaries. Moreover, each sinonasal tumor exhibits distinct growth and infiltration patterns, and the limited working space can restrict surgical visibility, particularly in the presence of bleeding [31,32,33,34]. In addition to the orbit and brain, the presence of multiple surrounding neurovascular structures, including the pterygopalatine fossa, infratemporal fossa, and upper parapharyngeal space, complicate achievement of clear margins and minimization of morbidity [35].

Extensive efforts have been made to minimize unnecessary resections while achieving safe tumor eradication, paralleling technological advances. Real-time navigation enhanced by 3D rendering, can improve precision in achieving adequate margins by roughly 20%, particularly in sinonasal endoscopic surgery [28,36]. The development of additive manufacturing technologies, allows precise fabrication of complex structures through a layer-by-layer approach [37]. These 3D printed models, derived from patient CT scans, are increasingly used in midface surgeries, including maxillectomy, to assess tumor conditions and bony anatomy [38]. This method not only facilitates the simulation of osteotomies and serves as a precise guide during resections, but also helps plan the type and size of the flap needed for reconstruction [39,40]. Additionally, it allows the manufacture of titanium mesh plates for orbital floor reconstruction or plates for fixing free vascularized bone grafts, which can decrease surgical times [41,42,43,44].

Despite advancements in technology, research on the role of 3D printing in improving negative resection margins, a key prognostic factor for oncologic outcomes, remains limited. This study is a retrospective analysis of 42 patients who underwent conventional maxillectomy versus 3D printing-guided maxillectomy at a single center to evaluate the role of 3D printing in oncological outcomes.

The introduction of 3D printing is relatively recent, and the prevalence of sinonasal cancer is low, limiting the number of patients included in this study [11,45]. Though significant results were not achieved, the 3D printing-guided group (81.3%) demonstrated a superior negative resection margin status compared with the conventional group (76.9%). Despite a higher proportion of T3 and T4 stages in the 3D printing-guided group (75.1% vs. 50% in the conventional group), the superior outcomes suggest the benefits of simulation for achieving adequate margins.

However, the 3D printing-guided group exhibited an 18.7% rate of positive resection margins. Because 3D printing is outsourced rather than produced in-hospital, the patient CT scans used for 3D printing are often obtained 4 to 6 weeks prior to surgery. The tumor may grow during this period, potentially leading to discrepancies between the 3D model and the actual surgical condition. However, in our study, positive resection margins were observed in the posterior maxillary wall, a region not covered by the 3D printing guidance. Additionally, to minimize discrepancies between CT imaging and surgical findings, contrast-enhanced CT scans were performed for patients whose surgeries occurred more than four weeks after the initial CT imaging. Additionally, 3D printing cannot account for microscopic infiltration, which may not be visible on the CT scan and could result in underestimation of actual tumor extent. These factors limit analysis of margin resection even with the assistance of 3D printing.

In contrast to the resection margin results, LVI was more prevalent in the 3D printing-guided group compared with the conventional group. The risk of LVI increases with T stage, and the higher proportion of advanced T stages (T3 and above) in the 3D printing-guided group may account for this unfavorable outcome [3]. Generally, PNI is more common in adenoid cystic carcinoma than in SCC [46]. A higher rate of PNI can be expected in the 3D printing-guided group, which has a higher proportion of adenoid cystic carcinoma. However, this study found a lower, albeit not significantly different, rate of PNI in the 3D printing-guided group.

The 3D printing-guided group, which achieved superior resection margin results, also demonstrated improved outcomes in 5-year local recurrence-free survival, 5-year recurrence-free survival, and 5-year overall survival, compared with the conventional group, although these findings were not significant. This aligns with other studies that emphasize the importance of resection margins as a critical prognostic factor [28].

This study has several limitations. As a retrospective study with a small sample size, it was challenging to control patient characteristics such as tumor histology and TNM stage. While the 3D printing-guided group showed superior results in resection margins and oncological outcomes compared with the conventional group, the small number of patients limited significant conclusion. Future prospective studies with larger cohorts are needed to confirm these findings, and it would also be beneficial to compare and analyze cosmetic and functional outcomes following reconstruction using 3D printing.

## 5. Conclusions

The study underscores the potential of 3D printing technology in enhancing surgical precision, particularly in achieving negative resection margins during maxillectomy, although these improvements were not significant compared to conventional methods. Despite showing a trend toward superior oncological outcomes in terms of recurrence-free and overall survival rates, the limited sample size and retrospective nature of the study highlight the need for larger, prospective studies to confirm these findings.

## Figures and Tables

**Table 1:** Patient and tumor characteristics.

Covariate	Overall *N* = 42	3D Printing-Guided Maxillectomy *n* = 16	Conventional Maxillectomy *n* = 26	*p*-Value
Sex (%)				0.055
male	22 (52.4)	5 (22.7)	17 (77.3)	
female	20 (47.6)	11 (55.0)	9 (45.0)	
Age, mean (SD)	56.6 ± 2.4	62.1 ± 17.3	53.8 ± 13.8	0.092
Tumor histology				0.532
Squamous cell carcinoma	28 (66.7)	9 (56.3)	19 (73.1)	
Adenoid cystic carcinoma	12 (28.6)	6 (37.5)	6 (23.1)	
Adenocarcinoma	2 (4.7)	1 (6.3)	1 (3.8)	
T stage (%)				0.453
T1:T2:T3:T4	4:13:11:14 (9.5:31.0:26.2:33.3))	1:3:5:7 (6.3:18.8:31.3:43.8)	3:10:6:7 (11.5:38.5:23.1:26.9)	
N stage (%)				0.437
N0:N1:N2:N3	38:1:2:1 (90.5:2.4:4.8:2.4)	16:0:0:0 (100:0:0:0)	22:1:2:1 (84.6:3.8:7.7:3.8)	
Type of maxillectomy (Brown classification)				
Ia	1 (2.4)	0 (0)	1 (3.8)	0.543
Ib	0 (0)	0 (0)	0 (0)	
Ic	0 (0)	0 (0)	0 (0)	
Id	0 (0)	0 (0)	0 (0)	
IIa	0 (0)	0 (0)	0 (0)	
IIb	5 (11.9)	1 (6.3)	4 (15.4)	
IIc	0 (0)	0 (0)	0 (0)	
IId	1 (2.4)	1 (6.3)	0 (0)	
IIIa	0 (0)	0 (0)	0 (0)	
IIIb	17 (40.5)	7 (43.8)	10 (38.5)	
IIIc	0 (0)	0 (0)	0 (0)	
IIId	4 (9.5)	1 (6.3)	3 (11.5)	
IVa	0 (0)	0 (0)	0 (0)	
IVb	1 (2.4)	1 (6.3)	0 (0)	
IVc	0 (0)	0 (0)	0 (0)	
IVd	1 (2.4)	1 (6.3)	0 (0)	
V	2 (4.8)	1 (6.3)	1 (3.8)	
VI	10 (23.8)	3 (18.8)	7 (26.9)	
Type of reconstruction				0.523
None	8 (19.0)	3 (18.8)	5 (19.2)	
Anterolateral flap	25 (59.5)	10 (62.5)	15 (57.7)	
Fibular flap	1 (2.4)	1 (6.3)	0 (0)	
Skin graft	8 (19.0)	2 (12.5)	6 (23.1)	

**Table 2:** Pathologic and treatment data and oncological outcomes between 3D printing-guided maxillectomy and conventional maxillectomy.

Covariate	Overall *N* = 42	3D Printing-Guided Maxillectomy *n* = 16	Conventional Maxillectomy *n* = 26	*p*-Value
Final resection margin negative: positive, *n* (%)	33:9 (78.6:21.4)	13:3 (81.3:18.7)	20:6 (76.9:23.1)	0.720
Lymphovascular invasion yes: no: unknown, *n* (%)	28:7:6 (68.3:17.1:14.6)	12:1:2 (80.0:6.7:13.3)	16:6:4 (61.5:23.1:15.4)	0.367
Perineural invasion yes: no: unknown, *n* (%)	25:10:7 (61.0:24.4:14.6)	9:5:2 (56.2:31.3:12.5)	16:6:4 (61.5:23.1:15.4)	0.960
Initial treatment, *n* (%)				0.370
Surgery only	12 (28.6)	5 (31.3)	7 (26.9)	
Surgery + adj. RT	30 (71.4)	11 (68.8)	19 (73.1)	
Oncologic outcomes, *n* (%)				
Local recurrence	9 (21.4)	2 (12.5)	7 (26.9)	0.240
Regional recurrence	3 (7.1)	0 (0)	3 (11.5)	0.275
Distant metastasis	11 (26.2)	4 (25.0)	7 (26.9)	1.000
Death	7 (16.7)	2 (12.5)	5 (19.2)	0.690
5-year local recurrence-free survival (%)		87.5	58.7	0.236
5-year recurrence-free survival		62.5	39.1	0.233
5-year overall survival		84.4	70.1	0.435

## Data Availability

The data presented in this study are available on request from the corresponding author due to the privacy of patients and medical centers.
